# Infected cephalhaematoma causing osteomyelitis: case report and literature review

**DOI:** 10.1093/jscr/rjac225

**Published:** 2022-05-27

**Authors:** Asfand Baig Mirza, Timothy Boardman, Samantha Ashworth, Wisam Al-Faiadh, Razna Ahmed, José Pedro Lavrador, Eleni Maratos, Chris Chandler, Cristina Bleil, Bassel Zebian

**Affiliations:** Department of Neurosurgery, King’s College Hospital NHS Foundation Trust, London, UK; GKT School of Medical Education, King’s College London, London, UK; GKT School of Medical Education, King’s College London, London, UK; Department of Neurosurgery, King’s College Hospital NHS Foundation Trust, London, UK; GKT School of Medical Education, King’s College London, London, UK; Department of Neurosurgery, King’s College Hospital NHS Foundation Trust, London, UK; Department of Neurosurgery, King’s College Hospital NHS Foundation Trust, London, UK; Department of Neurosurgery, King’s College Hospital NHS Foundation Trust, London, UK; Department of Neurosurgery, King’s College Hospital NHS Foundation Trust, London, UK; Department of Neurosurgery, King’s College Hospital NHS Foundation Trust, London, UK

## Abstract

Infected cephalhaematomas are rare and can lead to complications such as sepsis, meningitis and osteomyelitis. We present an infected cephalhaematoma in a neonate with resultant underlying osteomyelitis and a review of the literature. Our patient presented 6 days following birth with a fever and a swelling consistent with cephalhaematoma. He was managed with intravenous antibiotics and early surgical intervention. Imaging demonstrated underlying osteomyelitis. The patient made a full recovery and was discharged home on completing his antibiotic course. On reviewing the literature, it is clear that early diagnosis and treatment with surgical intervention and antibiotic therapy are associated with improved outcome and can reduce the possibility of osteomyelitis developing.

## INTRODUCTION

**Figure 1:**
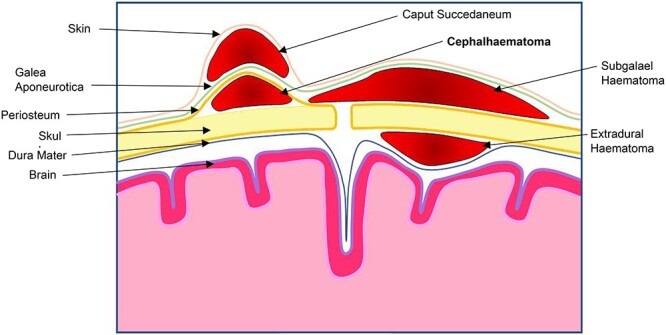
Drawing of scalp demonstrating tssshe locations of common haematomas of the scalp in relation to the different layers.

Neonatal cephalhaematomas result from subperiosteal haemorrhage (Fig. 1). They occur in 1–2% of all births [1] and are far more common in instrumented deliveries [2], although they can also occur following non-instrumented deliveries, caesarean sections [3] and the use of scalp electrodes [4]. They are the most common finding following birth trauma [5]. Despite this, cephalhaematomas are largely benign and the vast majority will resolve within a few weeks without any intervention [6]. On occasion, however, they can become infected. The appearance of local and systemic signs of infection should raise the alarm and prompt investigation [7]. By far the most common organism responsible for infected cephalhaematomas is *Escherichia coli* (*E. coli*; [3, 4, 7–12]) followed by a number of cases of *Staphylococcus aureus* infection [13, 14] and other bacterial pathogens including *Enterococcus faecalis* and *Bacteroides fragilis* [3].

Infected cephalhaematomas have the potential to lead to further complications such as sepsis, osteomyelitis and/or meningitis [12], although this is rare. The development of osteomyelitis in infected cephalhaematomas may be due to local haematogenous spread [3, 4]. In this paper, we present a neonate who was diagnosed with a large left-sided infected cephalhaematoma with resultant osteomyelitis. The child made a full recovery following a course of intravenous antibiotics and surgical intervention. Osteomyelitis underlying an infected cephalhaematomas fortunately remains rare.

## CASE PRESENTATION

A 6-day-old male was admitted to the Emergency Department with fever (38°C) in addition to a swelling on his head, which had been present since birth. The mother had been induced at 38 weeks due to maternal diabetes. The child was delivered by forceps after an unsuccessful attempt at vacuum extraction but was in good condition at birth (Appearance, Pulse, Grimace, Activity, Respiration 10, weight 2.96 kg). A large cephalhaematoma was apparent on the left side of the head confined to the parietal bone with an overlying abrasion. The child was otherwise well and mother and child were discharged home after delivery.

**Figure 2:**
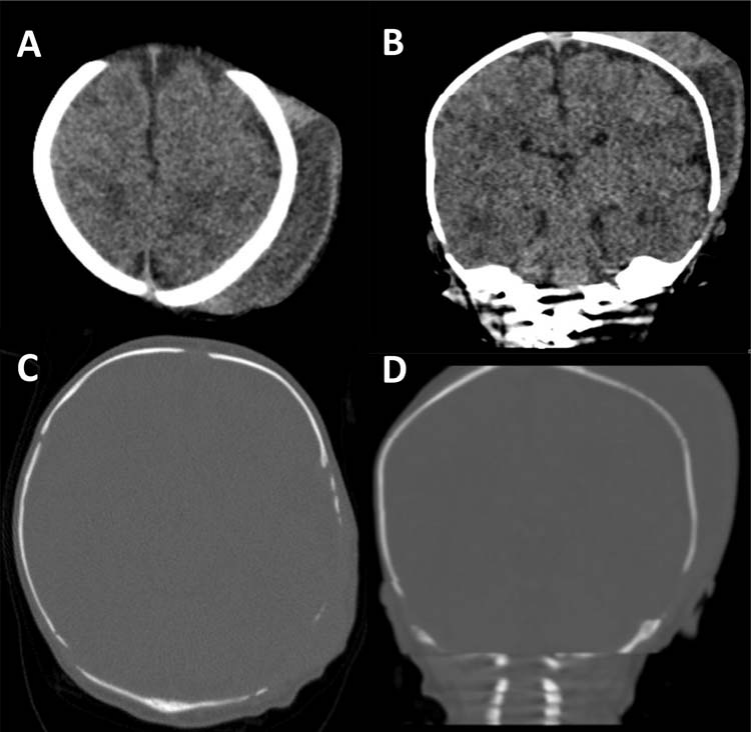
CT scan of our patient. In (**A**) and (**B**) the size of the cephalhaematoma can clearly be seen. In (**C**) and (**D**) the thinned, moth-eaten appearance of the left parietal region is well demonstrated.

On assessment in the Emergency Department a septic screen revealed a raised C-reactive protein (CRP) of 291 and normal urine dip, chest X-ray and lumbar puncture. He was admitted and started on Benzylpenicillin and Gentamicin. A computed tomography (CT) was performed demonstrating a large collection over the left parietal bone with a moth-eaten appearance of the underlying skull suspicious of osteomyelitis (Fig. 2).

**Figure 3:**
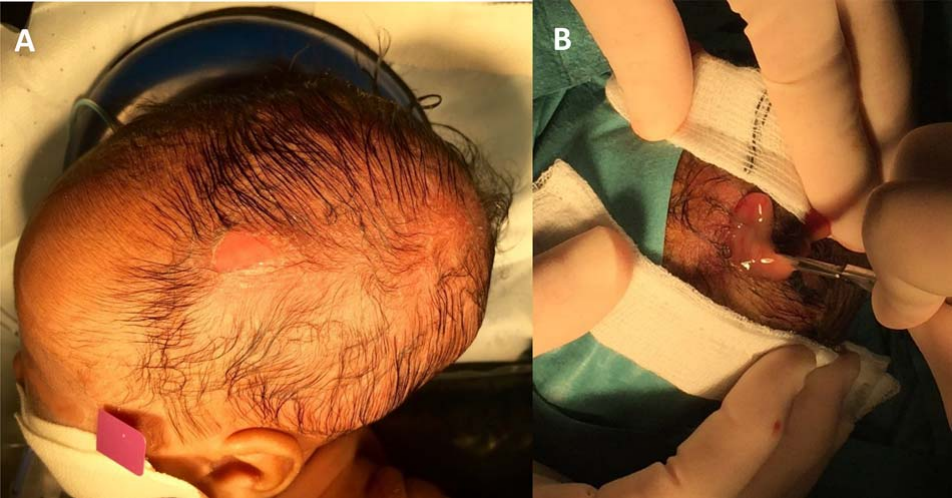
Photos illustrating the clinical stigmata of infection. In (**A**) the region of the infected cephalhaematoma is defined by erythema and an overlying abrasion. In (**B**) incision is made and the purulent collection seen.

**Figure 4:**
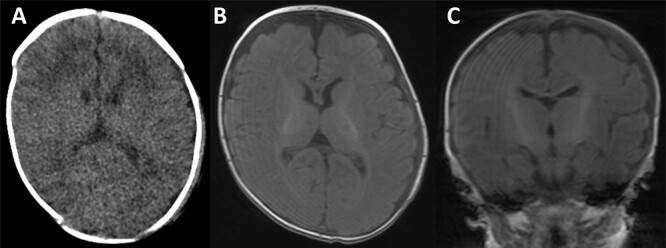
CT scan and MRI taken of the patient upon discharge. In all images, a well-defined skull with no thinning is illustrated and with a resolved haematoma.

The collection was tapped, and pus was aspirated and sent for microscopy, culture and sensitivities. Gram negative rods were seen in the aspirate and a surgical evacuation was performed. A left parietal incision over the dome of the collection was performed, the pus evacuated and copious irrigation with warm Ringer’s solution and hydrogen peroxide was performed, followed by debridement of the space and underlying bony surface (Fig. 3). *Escherichia coli* was cultured from both pus and bone samples and *S. epidermidis* was cultured from the blood. Following the procedure, the patient improved with no further fevers and with a progressive decrease in CRP. He received 3 weeks of intravenous antibiotics followed by oral ciprofloxacin for a week. He was discharged home 20 days after surgery. Repeat CT and magnetic resonance imaging (MRI) of the head revealing no complications (Fig. 4).

## DISCUSSION

In the vast majority of cases, cephalhaematomas will resolve naturally without any need for treatment [15]. However, if infection is suspected, timely investigation and management are essential.

Aspiration should be performed for both diagnostic purposes and therapeutic effect in any case of suspected infected cephalhaematoma [16]. There has been one case report where an infected cephalhaematoma was diagnosed based on MRI findings alone without diagnostic aspiration [17], however, there is simply not enough evidence to recommend it over aspiration, which is considered the gold standard. Once the offending pathogens are confirmed, antibiotics should be administered according to sensitivities. Fortunately, in most cases of infected cephalhaematoma with underlying osteomyelitis, there is usually only one causative pathogen and thus antibiotics can be tailored appropriately. Currently there is no definitive duration of treatment in the literature, however, it has been suggested that ideally antibiotics should be given for 4–6 weeks if underlying osteomyelitis is suspected and for 3 weeks for meningitis and bacteraemia alone [12]. Shorter antibiotic treatment duration and delayed diagnosis has been associated with late meningitis [10, 18]. CT, MRI or scintigraphy have been used and recommended to confirm potential osteomyelitis [19].

In 13 out of 19 cases reported in the literature [4, 7–9, 12, 13, 20–25], drainage and antibiotics were sufficient for managing an infected cephalhaematoma with underlying osteomyelitis. However, in many cases, debridement [3, 10, 11, 14, 26] and irrigation [3] have been advocated. By tapping in the early stages of a suspected infected cephalhaematoma, prompt treatment can be initiated with reduction in delayed complications [19]. Of note, tapping itself has been associated with infection so clinical judgement should guide its indication.

In conclusion, despite the rarity of infected cephalhaematomas with underlying osteomyelitis, with swift, appropriate intervention, the outlook is generally good for neonates and the vast majority make a full recovery over the following weeks.

We advocate early diagnosis and surgical evacuation to prevent local (osteomyelitis) and systemic (meningitis and sepsis) complications, as disease progression has been reported on antibiotics alone [10].

## CONFLICT OF INTEREST STATEMENT

None declared.
